# High expression levels of MAGE-A9 are correlated with unfavorable survival in lung adenocarcinoma

**DOI:** 10.18632/oncotarget.6741

**Published:** 2015-12-23

**Authors:** Xiaolu Zhai, Liqin Xu, Siya Zhang, Huijun Zhu, Guoxin Mao, Jianfei Huang

**Affiliations:** ^1^ Department of Chemotherapy, Nantong University Affiliated Hospital, Nantong 226001, Jiangsu, China; ^2^ Department of Respiratory, Nantong University Affiliated Hospital, Nantong 226001, Jiangsu, China; ^3^ Department of Pathology, Nantong University Affiliated Hospital, Nantong 226001, Jiangsu, China

**Keywords:** MAGE-A9, lung adenocarcinoma, immunohistochemistry, prognosis, apoptosis

## Abstract

A variety of melanoma-associated antigen-A (MAGE-A) protein are commonly detected in lung cancers. Their biological function is not well characterized but may involve cell cycle progression and the regulation of apoptosis. We hypothesized that MAGE-A9 is involved in the regulation of apoptosis. To test this hypothesis, we evaluated MAGE-A9 protein expression by immunohistochemical staining and we assessed the relationship between the expression of MAGE-A9 and clinical pathological parameters. In addition, we investigated the effect of MAGE-A9 down-regulation in lung adenocarcinoma. The results showed that a high expression level of MAGE-A9 protein in lung adenocarcinoma tumor cells was related to larger tumor diameter (*P* = 0.013) and poor differentiation (*P* = 0.029). Cox regression analysis revealed that the expression of MAGE-A9 in lung adenocarcinoma tumor cells (*P* < 0.001) is an independent prognostic factor in five-year survival rates. NSCLC cells with silenced MAGE-A9 had decreased cell proliferation, migration and invasion in cell culture compared to corresponding control cells. The NSCLC cells showing down-regulated MAGE-A9 induced the expression of apoptosis-associated proteins. In addition, MAGE-A9 was associated with resistance to conventional chemotherapeutic agents. Our findings provide evidence that MAGE-A9 could be a potential therapeutic target in NSCLC.

## INTRODUCTION

Lung cancer is the most frequently occurring type of cancer and is the leading cause of cancer deaths worldwide. Lung cancer can be divided into two types: non-small cell lung cancer (NSCLC) and small cell lung cancer (SCLC) [1–3]. The prognosis for NSCLC patients is highly dependent on the stage at diagnosis. Despite efforts to develop early screening tools, a majority of tumors are detected at an advanced stage, and approximately 85% of these cancers are identified as NSCLC [3–4]. Although multi-model treatment strategies including surgery, chemotherapy, radiotherapy and immunotherapy are used, the prognosis of these patients remains poor, with a 5-year overall survival rate of approximately 10% and a median survival time of 16 to 18 months [2, 5]. With recent developments in gene transfer technology, gene-targeted therapy is expected to become the good treatment for NSCLC [6]. Therefore, it is necessary to explore novel biological molecular markers for predicting the progression of NSCLC and aiding targeted therapy.

The MAGE (melanoma-associated antigen) gene is part of a group of tumor-associated antigens and was the first discovered for melanoma. The family is composed of genes that all share a homologous MAGE-conserved domain of approximately 200 amino acids. In humans, the family contains 37 protein-coding genes. Based on differences in tissue-specific gene expression and gene structure, MAGE genes are classified as type I (MAGE-A, MAGE-B, and MAGE-C) and type II (MAGE-D, MAGE-E, MAGE-F, MAGE-H, MAGE-L and NDN) genes [7–8]. Type II MAGEs are almost universally expressed in normal tissues and cancer cells [9–10]. Type I MAGE genes expression is restricted to a small number of normal tissues such as spermatocytes, placenta, and certain stages of embryonic development [9–10]. Type I MAGE genes are silent or expressed at low levels in normal adult tissues but are re-expressed in selected tumor types [11–12]. Because of this particular expression pattern, type I MAGE genes are categorized as members of the cancer/testis antigen (CTAg) gene family [13–15]. Its potential immunogenicity offers possible research directions that may lead to a therapeutic vaccine [16–19]. Because MAGE genes are not expressed in normal adult tissues, except in testes and placenta, where they are expressed in a large variety of neoplastic lesions, MAGE genes are considered tumor-specific antigens and ideal targets for cancer immunotherapy [20–21]. These antigens are presented on the cell surface by MHC-II molecules and identified by cytotoxic T lymphocytes (CTLs). Therefore, this is an ideal target molecule for CTL mediated-specific immune therapy [22–23].

MAGE-A is a multigene family consisting of 12 homologous genes MAGE-A1 to MAGE-A12 located at chromosome Xq28 [15, 24]. Studies have shown some of the MAGE-A genes are expressed in NSCLC and have become targets for lung cancer immuno-therapy [25–27]. MAGE-A9 is frequently expressed in urinary tumors and can provide prognostic information in bladder cancer and renal cell carcinoma. In addition, MAGE-A9 is overexpressed in laryngeal squamous cell carcinoma, cutaneous T-cell lymphomas, esophageal adenocarcinomas, and hepatocellular carcinoma [28–34]. To the best of our knowledge, MAGE-A9 expression in lung adenocarcinoma and its correlation with clinical parameters have not been evaluated to date. Thus, we detected MAGE-A9 expression in lung adenocarcinoma samples to analyze the associations between MAGE-A9 expression and clinicopathologic data in a group of patients with lung adenocarcinoma. In addition, we explored the effect of MAGE-A9 silencing *in vitro*.

## RESULTS

### The expression of MAGE-A9 in lung adenocarcinoma by IHC analysis

**Figure 1:**
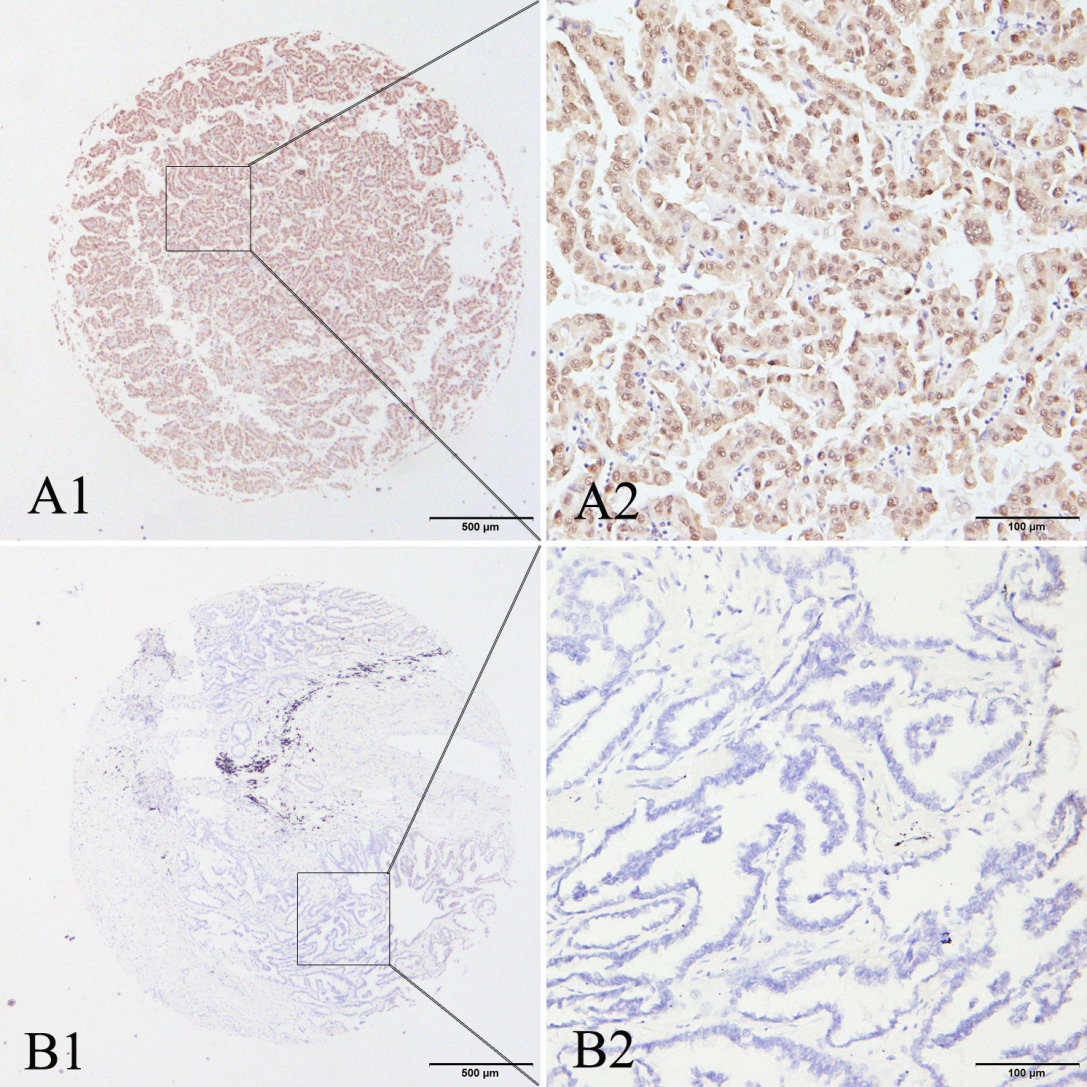
Representative patterns of MAGE-A9 protein expression in lung adenocarcinoma and adjacent noncancerous tissues (**A1**) and (**A2**) show positive staining in the cytoplasm and nucleus. (**B1**) and (**B2**) show a negative IHC reaction. Original magnification: (A1–B1) ×40; and (A2–B2) ×400.

Our previous research showed high expression of MAGE-A9 in tumor and stromal cells of non-small cell lung correlates with poor survival [35]. Here we performed IHC analysis to examine MAGE-A9 expression in lung adenocarcinoma. Positive staining for MAGE-A9 was mainly localized to tumor cells in the cytoplasm and the nucleus at different levels. High MAGE-A9 expression was detected in 42.78% of lung adenocarcinoma tissues (77/180) compared to 22.34% of matched tumor-adjacent tissues (21/94) (χ^2^ = 11.226, *P* = 0.001). Typical IHC staining patterns for MAGE-A9 in lung adenocarcinoma are shown in Figure 1.

### The association between MAGE-A9 expression and clinicopathological parameters

The relationship between high MAGE-A9 expression and the clinicopathological features in 180 cases of lung adenocarcinoma are shown in Table 1. The high expression of MAGE-A9 in lung adenocarcinoma tumors was significantly associated with differentiation (χ^2^ = 4.759, *P* = 0.029) and tumor diameter (χ^2^ = 6.205, *P* = 0.013). By contrast, no statistically significant association was found for gender, age, lymph-node metastasis and TNM Classification.

**Table 1:** Association of MAGE-A9 expression with the clinical characteristics of lung adenocarcinoma

Characteristic	*n*	Low or no expression (%)	High expression (%)	Pearson *χ^2^*	*P* value
Total	180	103 (57.22)	77 (42.78)		
Gender					
Male	91	46 (50.55)	45 (49.45)	3.347	0.067
Female	89	57 (64.04)	32 (35.96)		
Age					
≤ 60 years	82	48 (58.54)	34 (41.46)	0.106	0.744
> 60 years	98	55 (56.12)	43 (43.88)		
Differentiation					
Well and Moderately	156	95 (60.90)	61 (39.10)	4.759	0.029*
Poorly	22	8 (36.36)	14 (63.64)		
Unknown	2	0	2		
Tumor size					
≤ 3 cm	101	66 (65.35)	35 (34.65)	6.205	0.013*
> 3 cm	79	37 (46.84)	42 (53.16)		
Lymph node metastasis					
Yes	71	37 (52.11)	34 (47.89)	1.167	0.280
No	101	61 (60.40)	40 (39.60)		
Unknown	8	5	3		
TNM Classification					
I	107	68 (63.55)	39 (36.45)	4.760	0.093
II	43	22 (51.16)	21 (48.84)		
III and IV	30	13 (43.33)	17 (56.67)		

* *P* < 0.05.

### Survival analysis

**Figure 2:**
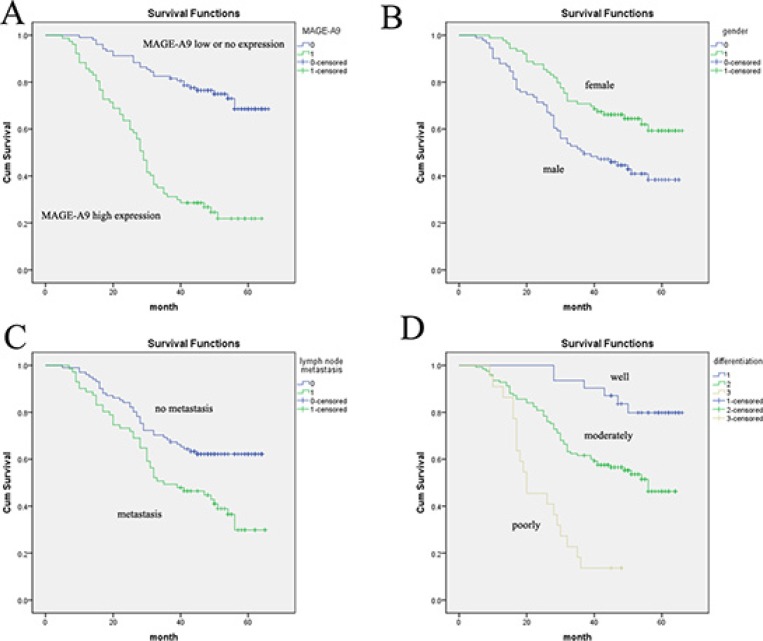
Analysis of lung adenocarcinoma patient survival using the Kaplan-Meier method (**A**) The high MAGE-A9 expression group (green line) has a significantly lower survival rate than the low/no MAGE-A9 expression group (blue line). (**B**) Overall survival in female cases was significantly longer than in male cases. (**C**) Overall survival in cases with no lymph-node metastasis was significantly longer than in cases with lymph-node metastasis. (**D**) Survival curves based on differentiation. Differentiation = 1 is the well-differentiated group (blue line); differentiation = 2 is the moderately differentiated group (green line); differentiation = 3 is the poorly differentiated group (brown line).

Several known predictive factors of poor outcome in NSCLC were assessed to confirm that our cohort of patients was representative of those with NSCLC (Table 2). As expected, MAGE-A9 protein overexpression (*P* < 0.001) was significantly associated with 5-year survival by Cox regression univariate analysis. In addition, correlations with other prognostic factors such as gender (*P* = 0.002), tumor diameter (*P* = 0.001), differentiation (*P* = 0.001), lymph node metastasis (*P* = 0.002) and TNM classification (*P* = 0.001) were also statistically significant. All of these factors were included in a multivariable analysis. High MAGE-A9 expression in tumor tissue (*P* < 0.001), male (*P* = 0.007), poor differentiation (*P* = 0.001) and lymph node metastasis (*P* = 0.037) were identified as independent predictive factors in the poor outcome of lung adenocarcinoma. Kaplan-Meier survival curves again showed that high MAGE-A9 expression corresponded with a significantly shorter survival time compared with those showing no or low MAGE-A9 protein expression (Figure 2).

**Table 2:** Univariate and multivariable analyses of prognostic factors in lung adenocarcinoma for 5-year survival

Variable	Univariate analysis			Multivariate analysis		
	HR	*P* value	95% CI	HR	*P* value	95% CI
MAGE-A9 expression						
High vs low	4.728	0.001*	2.989−7.477	3.356	0.001*	2.093−5.380
Gender						
Male vs female	0.510	0.002*	0.330−0.788	0.533	0.007*	0.337−0.845
Age (years)						
≤ 60 vs > 60	1.308	0.220	0.851−2.010			
Tumor size (cm)						
≤ 3 vs > 3	2.575	0.001*	1.669−3.972	1.616	0.066	0.968−2.700
Differentiation						
Well and mod vs poorly	3.422	0.001*	2.247−5.211	2.780	0.001*	1.741−4.439
Lymph node metastasis						
Yes vs no	1.964	0.002*	1.274−3.027	1.757	0.037*	1.035−2.983
TNM classification						
stage I vs stage II vs stage III/IV	1.461	0.001*	1.163−1.835	0.907	0.590	0.636−1.293

* *P* < 0.05; CI, confidence interval; HR, hazard ratio.

### The association of MAGE-A9 expression with NSCLC

**Figure 3:**
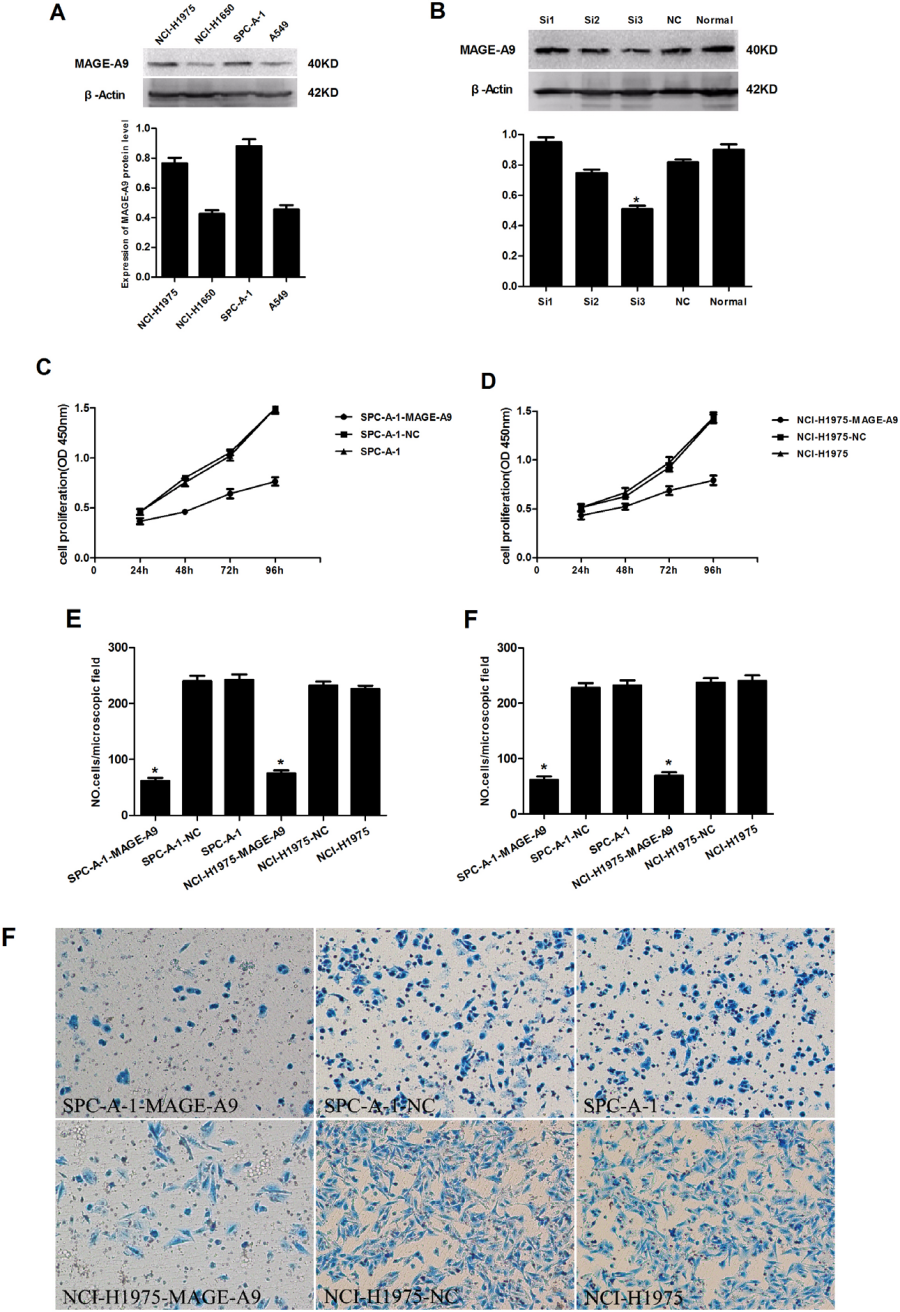
The effects of depleting the expression of MAGE-A9 on cell proliferation, migration and invasiveness of lung carcinoma cells (**A**) MAGE-A9 protein expression in four NSCLC cell lines. β-Actin was used as a loading control. (**B**) Western blots were used to select the most effective silencing siRNA for targeting human MAGE-A9. (**C**) and (**D**) The proliferation ability of the two experimental cell lines was examined using CCK-8 at 450 nm. Specifically, 5 × 10^3^ cells were seeded in 100 μL of medium per well into 96-well plates (three wells per each group). Then, 10 μL of CCK8 solution was added to the culture medium in each well after 24 h, 48 h, 72 h and 96 h. The cells were then incubated for an additional 3 h. The absorbance was determined at a wavelength of 450 nm. (**E**) Migration and (**F**) invasion ability were presented as the total number of cells that migrated to the bottom chamber without or with the Transwell precoated with Matrigel, as calculated in at least six random fields (total magnification ×200) per filter. The lower “F” is photo of six random fields represent invasion ability. It is corresponding to upper “F”. (*P < 0.05).

We examined MAGE-A9 protein expression in four NSCLC cell lines (SPC-A-1, A549, NCI-H1975 and NCI-H1650). Western blot results showed higher expression of MAGE-A9 in the SPC-A-1 and NCI-H1975 cell lines than in the A549 and NCI-H1650 cell lines (Figure 3A).

### The effect of decreased MAGE-A9 expressionon cell proliferation, migration and the invasiveness of lung carcinoma cells

We explored the functional consequence of altering the expression of MAGE-A9 in NSCLC cell lines by examining three different sequences of siRNA targeting human MAGE-A9 and negative control siRNA. Western blot analysis identified MAGE-A9#3 as the most potent sequence for silencing (Figure 3B). We transfected MAGE-A9#3 siRNA into SPC-A-1 and NCI-H1975 cell lines, which had shown high MAGE-A9 protein expression. The transfected cells were named SPC-A-1-MAGE-A9 and NCI-H1975-MAGE-A9, and the corresponding negative controls were named SPC-A-1-NC and NCI-H1975-NC.

Subsequently, we evaluated the cell proliferation, migration and invasion of the two experimental cell lines. Cell lines silenced with MAGE-A9 siRNA (SPC-A-1-MAGE-A9 and NCI-H1975-MAGE-A9) had lower proliferative abilities than the corresponding controls and normal controls (Figure 3C and 3D). In the migration and invasion assays, fewer SPC-A-1-MAGE-A9 and NCI-H1975-MAGE-A9 cells migrated through the membrane in the migration chamber with or without the Transwell-precoated Matrigel compared to corresponding control cells (Figure 3E and 3F), and the difference was statistically significant (*P* < 0.05). These results indicated that silencing of MAGE-A9 expression decreased the cell proliferation, migration and invasion of NSCLC cells.

### MAGE-A9 expression and apoptosis

**Figure 4:**
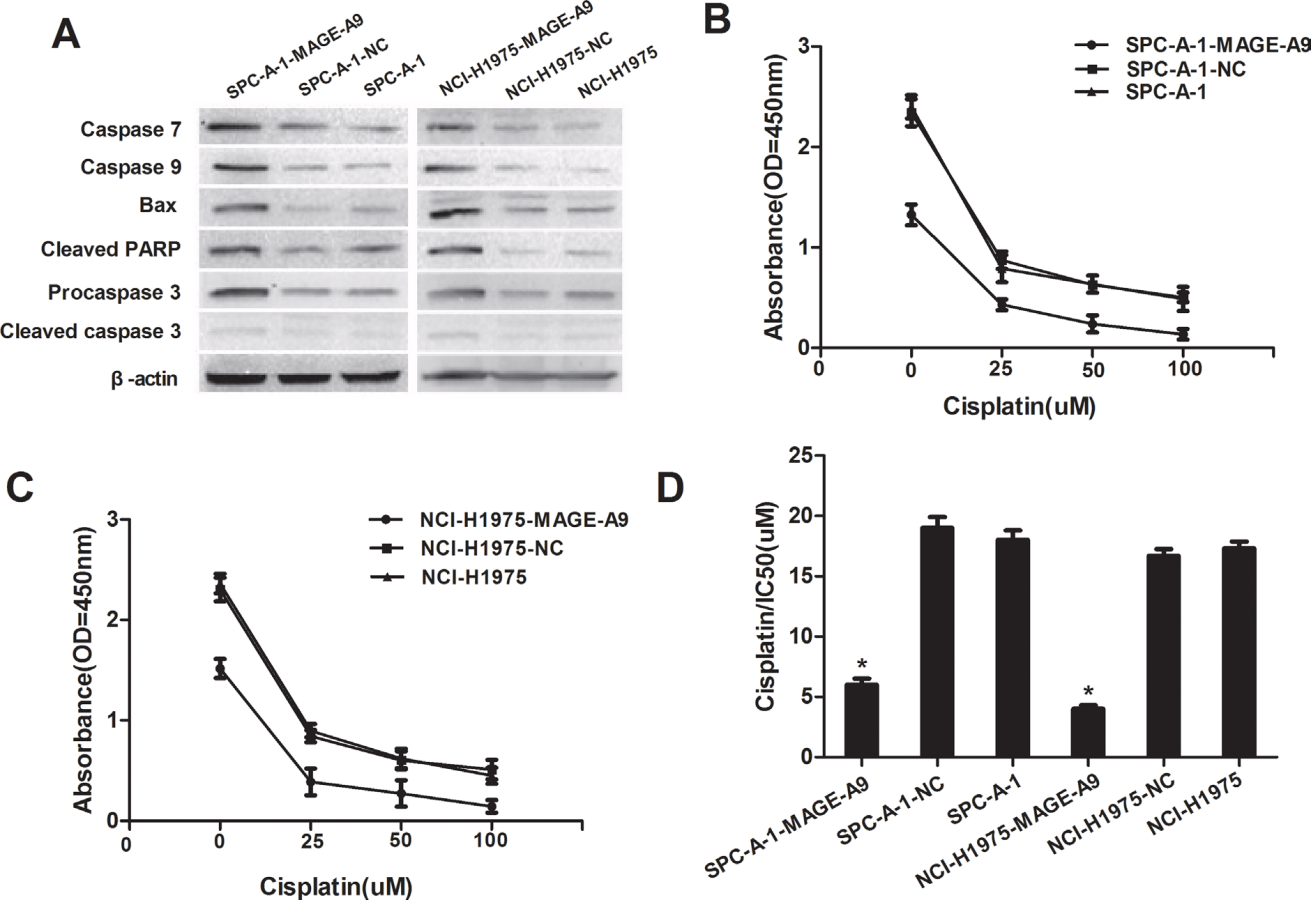
MAGE-A9 expression and apoptosis *in vitro* (**A**) Western blot analysis of apoptosis-associated protein expression in MAGE-A9 silenced cells. β-Actin was used as a loading control. (**B**) and (**C**) MAGE-A9 depletion in SPC-A-1-MAGE-A9 and NCI-H1975-MAGE-A9 cells contributed to an increased apoptotic response to cisplatin. (Error bars = 95% confidence interval, CI; *P* < 0.05). (**D**) Half-maximal inhibitory concentration (IC50) values of cisplatin. IC50 values were determined using CCK-8 as described above. (Error bars = 95% CI; *P < 0.05).

To explore the underlying mechanism by which MAGE-A9 induces lung tumor growth, apoptosis-associated protein expression was analyzed in MAGE-A9 silenced cells. Expressions of the pro-apoptotic protein Bax, Caspase-3, Caspase-7, Caspase-9 and Apoptosis Western Blot Cocktail were increased at different degrees in SPC-A-1-MAGE-A9 and NCI-H1975-MAGE-A9 cells, compared to corresponding control cells (Figure 4A). Our results suggest that silencing MAGE-A9 expression might promote apoptosis in NSCLC cells.

### MAGE-A9 overexpression confers resistance to chemotherapy-induced apoptosis

We investigated the association between MAGE-A9 expression and chemoresistance to four chemotherapeutic agents in NSCLC cells and explored whether MAGE-A9 plays a role in drug-induced apoptosis. MAGE-A9 depletion in SPCA-1-MAGE-A9 and NCI-H1975-MAGE-A9 cells contributed to an increased apoptotic response to various levels of chemotherapeutic reagents that are commonly used for lung cancer including GEM, DDP, Taxol and PEM (Figure 4B and 4C: *P* < 0.05, part data not shown). Specifically, the IC50 in SPC-A-1-MAGE-A9 and NCI-H1975-MAGE-A9 cells was lower than in control cells (Figure 4D *P* < 0.05). Hence, the down-regulation of MAGE-A9 might increase the sensitivity of NSCLC cells to chemotherapeutic reagents and may be related to our above demonstration that decreased MAGE-A9 expression could induce tumor cell apoptosis.

## DISCUSSION

Lung cancer is diagnosed at an advanced stage in most patients, which is the primary reason for the high mortality rate. Therefore, early detection of lung cancer is a major issue. New molecular targets for diagnosis and therapy are urgently needed to improve the prognosis for lung cancer patients. Recently, MAGE-A family members have been identified as promising immunotherapeutic targets for tumor therapy because they are strictly tumor specific [36–37]. Several basic clinical studies involving melanoma, esophagus cancer and lung cancer have utilized MAGE-A antigens and have shown encouraging results [38–40]. Although the normal physiological function of MAGE-A antigens remains unknown, their contribution to tumor development has been investigated recently.

It has been reported that MAGE expression is directly regulated by microRNAs, such as miR-34a, and that MAGE-A proteins can inhibit p53 function through direct and indirect mechanisms. The p53 tumor suppressor plays a protective in some tumors by coordinating changes in gene expression, which can lead to the elimination of cancer cells. Mage-A may block the association of p53 with its cognate sites in chromatin. Silencing of Mage-A expression leads to the up-regulation of several p53-responsive genes. These data reveal the novel mechanism by which Mage-A may suppress the p53 transcriptional program during tumor development [41]. MAGE-A inhibited apoptosis in proliferating malignancy cells through at least two novel mechanisms: inhibition of the up-regulation of Bax by a p53-dependent mechanism and maintenance of survivin expression by p53-dependent and independent mechanisms. Silencing of MAGE-A resulted in a loss of p53 ubiquitination and stabilization of p53 protein, thereby increasing the expression of Bax. Survivin was down-regulated in tumor cells after MAGE-A silencing regardless of the p53 status. These results strongly suggest that MAGE-A is an antagonist of p53-dependent pro-apoptotic transcriptional regulation [42].

In this present investigation, MAGE-A9 protein expression in lung adenocarcinoma tissues was evaluated using IHC, and the results showed that 42.78% of the cases exhibited high MAGE-A9 cytoplasmic and nuclear expression. Consistent with a previous study, our results support the hypothesis that cytoplasmic and nuclear MAGE-A9 expression is present in renal cell carcinoma [30]. Furthermore, we found that high MAGE-A9 expression in lung adenocarcinoma was significantly correlated with differentiation and tumor diameter. For survival analysis, our data clearly showed that high expression of MAGE-A9 is an independent predictive factor of poor outcomes in lung adenocarcinoma. These results were in agreement with previous studies in renal cell carcinoma [30] and bladder cancer [43]. A Kaplan-Meier analysis also verified that lung adenocarcinoma patients with high MAGE-A9 expression showed a significantly unfavorable life span.

Our *in vitro* findings shed light on how MAGE-A9 promotes NSCLC tumor progression. Four NSCLC cell lines were used to model the potential proliferative role of MAGE-A9 silencing in NSCLC. The functional effects of MAGE-A9 knockdown in two high-expression cell lines were consistent: decreased proliferation, migration and Matrigel invasion. These findings are consistent with recent studies, which report MAGE-A9 overexpression in breast cancer [44–45] and bladder cancer [43] and identify MAGE-A9-enhanced tumorigenicity and tumor growth. Although the physiological role of MAGE-A proteins remains largely unknown, it has been shown that DNA hypermethylation and histone deacetylation is the underlying mechanism responsible for MAGE-A9 gene silencing [44]. Increasingly, evidence suggests the involvement of DNA hypermethylation and histone deacetylation in the early carcinogenesis of the lung, involving the regulation of apoptosis and cell-cycle progression [46–47]. MAGE-A proteins are also more frequently expressed in chemotherapy (paclitaxel) resistance compared with chemotherapy-susceptible ovarian cancer, melanoma and multiple myeloma cell lines [48]. We observed that down-regulated MAGE-A9 expression promoted the sensitivity of four typical chemotherapeutic agents and inhibited the growth of cancer cells. These findings suggest that MAGE-A expression favors tumor-cell survival and that MAGE-A proteins function as oncoproteins. MAGE-A9 may play a key role in drug resistance in human cancer, and this likely occurs via multiple mechanisms. These data indicate that MAGE-A9 is critical for the survival of proliferating NSCLC cells and that MAGE-A9 is a promising therapeutic target for this disease. Hence, our study might open up a novel strategy for future cancer therapies through the modulation of cellular MAGE-A9 activities.

In summary, we confirmed the significant up-regulation of MAGE-A9 proteins in lung adenocarcinoma tissues and found that high expression levels are associated with poor prognosis. Moreover, we revealed that RNA interference of MAGE-A9 inhibited the proliferation, invasion and migration of NSCLC cell lines and restrained the tumorigenesis and development of NSCLC. Decreased MAGE-A9 expression could induce apoptosis as it occurs in NSCLC cells. Finally, MAGE-A9 might be associated with resistance to conventional chemotherapy, and down-regulation of MAGE-A9 may increase the sensitivity of chemotherapeutic reagents, which has important implications for cancer progression and treatment.

There are some limitations to this study. For example, overexpression of the MAGE-A9 gene has not been explored. Furthermore, the molecular mechanisms underlying MAGE-A9 involvement in cell proliferation, migration and chemosensitivity should be analyzed *in vivo*. If the results from further investigations support our findings, a strategy that targets MAGE-A9 might be expected to establish a high efficacy chemo-immunotherapeutic approach.

## MATERIALS AND METHODS

### Patients and resources

A panel of formalin-fixed, paraffin-embedded lung adenocarcinoma tissues (*n* = 180) and corresponding peritumoral normal tissues (*n* = 94) were excised from fresh surgical samples at the Affiliated Hospital of Nangtong University from 2004 to 2010. Clinical data (including age, gender, differentiation, tumor size, five-year follow-up survival records and other information) were obtained from the medical records of each patient. The average age of the group was 61.25 years (range 35–81 years). The 5-year actuarial overall survival was calculated from the date of surgery until the date of death or the last follow-up appointment. All patients were typed in accordance with the recent TNM stage classification system (UICC 2009). None of the patients received chemotherapy or radiotherapy prior to surgery. Representative 2.0 mm tissue cores from each sample were used to conduct TMA analysis (Shanghai Outdo Biotech, Shanghai, China). The study was approved by the Ethical Research Committee of the hospital.

### Immunohistochemistry

For immunohistochemical analysis, tissue sections were deparaffinized in 100% xylene and rehydrated in graded ethanol solutions. Antigen retrieval was performed by boiling in citrate buffer, pH 6.0, for 5 minutes in a pressure cooker. Sections were incubated with a primary anti-MAGE-A9 antibody (Abcam, Cambridge, UK)diluted 1:100 in TBS containing 1% bovine serum albumin at 4°C overnight and then incubated with anti-rabbit horseradish peroxidase-conjugated antibody at 37°C for 30 min. MAGE-A9 immunostaining was evaluated independently by two trained pathologists who had no knowledge of the clinical background of the cases. The positivity of cell staining was recorded as a percentage (0–100%). Staining intensity was graded on a scale of 0 (negative) to 3 (strong). The final MAGE-A9 staining score was a product of the intensity grading and the percentage of positive cells [49]. The cutoff point for the MAGE-A9 expression score that was statistically significant, in terms of overall survival (OS), was set using the X-tile software program (The Rimm Lab at Yale University, New Haven, CT) as described previously [50]. The degree of MAGE-A9 staining was quantified using a two-level grading system, and staining scores were defined as follows: 0–100, no expression to low expression; and 101–300, high expression.

### Cell lines and cell culture

Human NSCLC cell lines A549, SPC-A-1, NCI-H1975 and NCI-H1650 were purchased from the Type Culture Collection of the Chinese Academy of Sciences, Shanghai, China. All cell lines were maintained in RPMI-1640 medium (HyClone, Logan City, Utah, USA), supplemented with 10% fetal bovine serum (FBS), and cultured at 37°C in a humidified atmosphere containing 5% CO_2_.

### Western blot analysis

Total protein extracts from each cell line were obtained using a lysis buffer (Beyotime Institute of Biotechnology, China), and the protein concentration was determined using the BCA Protein Assay Kit with bovine serum albumin (BSA) as the standard (Thermo Scientific). Equal amounts of protein (20 μL per lane) were separated by SDS-polyacrylamide gel electrophoresis (PAGE) in 10% acrylamide gels and transferred to polyvinylidine difluoride (PVDF) membranes (Millipore Corporation, USA) at 300 mA for 2 h. The membrane was blocked in 5% fat-free milk and incubated with the following primary antibodies overnight at 4°C: mouse monoclonal anti-MAGE-A9 (1:300 dilution; Abcam), rabbit monoclonal anti-Bax (1:1000 dilution; Abcam), rabbit polyclonal anti-Caspase3 (1:300 dilution; Abcam), rabbit polyclonal anti-Caspase7 (1:1000 dilution; Abcam), rabbit monoclonal anti-Caspase9 (1:1000 dilution; Abcam) and Apoptosis Western Blot Cocktail (1:250 dilution; Abcam). The secondary antibodies were horseradish peroxidase (HRP)-conjugated goat anti-mouse antibody (1:1000, Proteintech group, USA) and horseradish peroxidase (HRP)-conjugated goat anti-rabbit antibody (1:1000, Proteintech group, USA). After stripping, the membrane was reprobed with β-actin (1:1000, Proteintech group, USA) overnight at 4°C, followed by incubation with a secondary antibody as stated above at room temperature for 2 h. Bands were visualized using an enhanced chemiluminescence system (ECL, Beyotime Institute of Biotechnology). The data were quantified by densitometry.

### SiRNA transfection

Three different siRNA sequences targeting human MAGE-A9 and negative control siRNA were designed by, and obtained from, Shanghai Invitrogen Corporation. The sequences of Si-MAGE-A9 were as follows: MAGE-A9#1 sense, 5′-GGUGGCUGAGUUGGUUCAUTT-3′ and antisense, 5′-AUGAACCAACUCAGCCACCTT-3′; MAGE-A9#2 sense, 5′-GCAAAGCCUCCGAGUUCAU TT-3′ and antisense, 5′-AUGAACUCGGAGGCUUUGC TT-3′; MAGE-A9#3 sense, 5′-CCAGCUAUGAGAAGGU CAUTT-3′ and antisense, 5′-AUGACCUUCUCAUAGCU GGTT-3′. Cells were then transfected with Lipofectamine^™^ 2000 (Invitrogen) according to the manufacturer's instructions.

### Cell proliferation assays

Cell proliferation was evaluated using the Cell Counting Kit-8 (CCK-8, Beyotime Institute of Biotechnology) according to the manufacturer's instructions. Briefly, the NSCLC cells, negative control cells and normal control cells (5 × 10^3^), in 100 μL of medium per well, were seeded into 96-well plates (three wells per each group); 10 μL of CCK8 solution was added to the culture medium in each well, and cells were incubated for 3 h. The absorbance was determined at a wavelength of 450 nm. The assays were repeated three times with triplicate samples.

### Transwell migration and invasion assays

For the analysis of cell invasion assays, modified Boyden chambers consisting of Matrigel-precoated Transwell membrane filter inserts with 8 μm pores were used in 24-well tissue culture plates (BD Biosciences, Bedford, MA). Cells from different groups (1 × 10^5^) were plated onto the top of the chamber in RPMI-1640 without FBS, and the bottom chamber was filled with RPMI-1640 containing 10% FBS as a chemoattractant. After 24 h of incubation in a 5% CO_2_ humidified chamber at 37°C, noninvading cells were removed by wiping the upper surface of the membrane with a cotton swab, and the filter membrane was fixed with 4% paraformaldehyde and stained with Exam MaSiLiang blue. The degree of invasion was quantified by counting the cells that had migrated through the membrane in at least six random fields (total magnification, ×200) per filter. Experiments were repeated three times in triplicate.

For cell migration, we used the modified Boyden chambers without the Matrigel-precoated Transwell membrane filter, using the method stated above.

### Chemotherapeutic cell treatments

Gemcitabine (GEM), cisplatin (DDP), paclitaxel (Taxol), and pemetrexed (PEM) were used at 0.1–100 μM to determine the half-maximal inhibitory concentration (IC50) values in cell lines, in which MAGE-A9 was silenced, and their corresponding controls cells. Cells (5 × 10^3^) were added to each well in a 96-well plate and cultured for 24 h. Cells were then treated with drugs for 12 h, and the medium was replaced by fresh medium without drugs for an additional 48 h. Cell viability was measured using a Cell Counting Kit-8 (Beyotime Institute of Biotechnology) at 450 nm as stated above. DMSO treatment was used as a control. The survival of each cell line was compared with their corresponding control cell line. Assays were repeated three times.

### Statistical analysis

Statistical analyses were performed using the SPSS V.20.0 software (SPSS Inc. Chicago, IL, USA) and the STATA V.9.0 software (Stata Corporation). χ^2^ tests were performed to evaluate whether MAGE-A9 expression was correlated with clinicopathological factors. For the TMA slides, the following clinical data were assessed: gender, age, tumor diameter, and other clinicopathological information. Survival curves were estimated using the Kaplan-Meier method. The factors shown to be of prognostic significance in the univariate models were evaluated in a multivariable Cox regression model. For all statistical analyses, *P*-values less than 0.05 were regarded as statistically significant. All statistical tests were two-sided.
